# Towards Systems Biology of Heterosis: A Hypothesis about Molecular Network Structure Applied for the Arabidopsis Metabolome

**DOI:** 10.1155/2009/147157

**Published:** 2008-08-27

**Authors:** Sandra Andorf, Tanja Gärtner, Matthias Steinfath, Hanna Witucka-Wall, Thomas Altmann, Dirk Repsilber

**Affiliations:** 1 Bioinformatics and Biomathematics Group, Genetics and Biometry Unit, Research Institute for the Biology of Farm Animals (FBN), Wilhelm-Stahl Allee 2, 18196 Dummerstorf, Germany; 2 Institute for Biochemistry and Biology, University of Potsdam, Karl-Liebknecht-Str. 24-25, 14476 Potsdam-Golm, Germany; 3 Institute for Genetics, University of Potsdam, Karl-Liebknecht-Str. 24-25, 14476 Potsdam-Golm, Germany

## Abstract

We propose a network structure-based model for heterosis, and investigate it relying on metabolite profiles from Arabidopsis. A simple feed-forward two-layer network model (the Steinbuch matrix) is used in our conceptual approach. It allows for directly relating structural network properties with biological function. Interpreting heterosis as increased adaptability, our model predicts that the biological networks involved show increasing connectivity of regulatory interactions. A detailed analysis of metabolite profile data reveals that the increasing-connectivity prediction is true for graphical Gaussian models in our data from early development. This mirrors properties of observed heterotic Arabidopsis phenotypes. Furthermore, the model predicts a limit for increasing hybrid vigor with increasing heterozygosity—a known phenomenon in the literature.

## 1. Introduction

"Biological function" is the core of biological research, but it is an ill-defined term. Geneticists, cellular biologists, structural biologists, biophysical chemists, and bioinformaticians all target different meanings in their respective research areas [1,2]. However, as a unifying notion, biological function always refers to *semantic* features and, as such, is always context-dependent. A specific state of any biological molecule alone is not accomplishing any biological function [3]. Rather, biological function resides in *interactions* [4–6]. The characteristics of such biological interactions, when analyzed on a genome-wide scale, are referred to as the *structure of biological networks* (including their dynamics). Relating structure of biological networks to biological function is therefore a major objective in biology, mirrored in recent developments such as systems biology.

A huge variety of biological networks exist; however, there are common characteristics. Biological network structure always arises as interaction of genetic determination and environmental influences, as well as internal systems dynamics. As pointed out by Somogyi and Sniegoski [5], interactions within specific representations of biological networks may either map directly to existing biomolecules, or may reflect rather indirect relations involving possibly many of hidden variables [7,8]. Most types of biological networks can be interpreted also as regulatory networks, in the sense that they "respond" to environmental or developmental challenges by changing their state or dynamics. A frequent approach to search for important network structures at a rather global level of biological networks is *statistical network modeling*. It starts out by screening for significant measures from graph theory [9–11]. Distributions of such measures can then be compared between biological, technical, and random networks, as well as between different classes of organisms [10,12,13], regimes of environmental challenges, or developmental periods [12]. If specific structures are discovered, their relation to a biological function of interest may be hypothesized and experimentally validated on further datasets.

**Figure 1:**
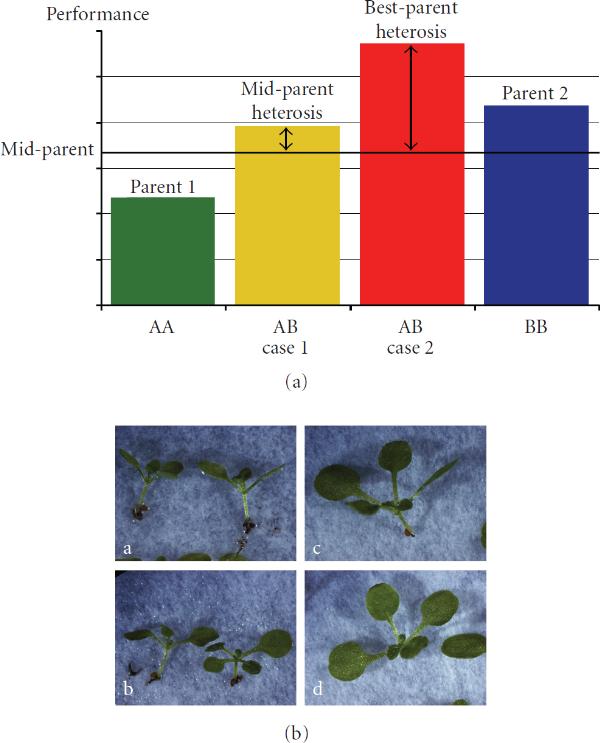
**Definition of heterosis**. (a) Quantitative genetics definition of midparent heterosis and best-parent heterosis (heterosis effect: arrows); (b) example from early development in *Arabidopsis thaliana*—cotyledon areas are the largest in heterozygous crosses (c, d) as compared to their homozygous parents (a, b).

In our case, we are interested in contributing to a systems biological understanding of the biological phenomenon of heterosis. Shull [14] defined the term heterosis as "increased vigor, size, fruitfulness, speed of development, resistance to disease and to insect pests, or to climatic rigors of any kind, manifested by crossbred organisms as compared with corresponding inbreds." See Figure 1(a) for a quantitative genetics definition of heterosis, and Figure 1(b) for an example of a trait showing a heterotic phenotype, cotyledon area in *Arabidopsis*. *Midparent heterosis* denotes an increase of performance relative to the mean of both parents, while *best-parent heterosis* describes the situation where the heterozygous offspring performs better than either parent. As early as 1952, Robertson and Reeve [15] suggested that heterozygotes are likely to possess a greater biochemical versatility by carrying a greater diversity of alleles. Heterosis would then result from a reduced sensitivity to environmental variations since in heterozygotes there will be additional ways of overcoming such challenges. In other words, the heterosis phenomenon may be due to higher adaptability in heterozygotes. On the genetic level, hypotheses explaining heterosis may be grouped into two groups. On one hand, dominant or overdominant modes of gene action are thought to play a major role, assuming recessive status for a majority of inferior alleles. On the other hand, enriched favourable epistatic interactions are discussed as the main reason for the heterosis phenomenon at the molecular level [16–18].

Gjuvsland et al. [19] demonstrate how epistatic interactions within statistical genetics models can be translated into functional structures of regulatory biological networks. In our contribution, we focus on these molecular network structures and ask the following question. Which structures of biological networks could systematically lead to higher adaptability in heterozygotes, and thus to the heterosis phenomenon? For investigating this question, we choose to follow a conceptual modeling approach [5,20,21]. Our model choice is based on a major result of statistical network modeling. Analyses of distributions of simple regulatory motifs both in prokaryotes and in eukaryotes point to similar results. The so-called *multi-input motif* is a significant and prominent part of regulatory biological networks [10,12,22]. The properties of networks of this type were studied by Steinbuch already in 1961 [23]. His studies were focusing on modeling and implementing models of associative learning. The so-called *Steinbuch matrix* is a two-layer feed-forward network. The information about which input vector is *associated* with which output vector is encoded within the pattern of presence/absence of connections between these two layers. We are going to use this Steinbuch network as a conceptual model for biological networks, and develop a hypothesis of heterosis based on biological network structure. We expect specific global structures in biological networks to be different between homozygotes and their heterozygous offspring.

To validate and further detail our network hypothesis of heterosis, we analyze partial correlation structures in experimental metabolite profile association networks from two different homozygous *Arabidopsis thaliana* lines and both reciprocal crosses as heterozygotes. These metabolite profiles were measured during early development of *Arabidopsis*, as during this time heterosis phenomena become manifest in this species [24]. We refer again to Somogyi and Sniegoski [5] following their argument that not only the transcriptome but also the metabolome could be viewed as a special mapping of the extended biological regulatory network. Such a mapping would include many indirect regulatory interactions involving hidden molecular variables which are part of other levels of gene expression.

Summarizing the objectives of our study, we motivate the proposal of a network structure-based hypothesis of heterosis, and look for heterozygote-specific network structures as predicted by a Steinbuch network conceptual modeling approach. Analyses of metabolite profiles of early development in *Arabidopsis thaliana* and further observations of heterosis in plants will serve as to validate and further adjust our hypothesis.

Section 2 describes the experimental dataset and our preprocessing prior to statistical network analyses. In Section 3, we describe our modeling approach as well as a small simulation study. Its results motivated our choice of network statistics for global assessment of network structures described in the remaining part of this section. The first part of Section 4 reports the simulation results. In its second part, we develop our network structure-based hypothesis of heterosis and its predictions. In the last part of this section, results of experimental data analysis as motivated by our model predictions are presented. Finally, in Section 5 we discuss the main findings of our study, along with their relevance and benefits, and constraints of our approach as well as future prospects.

## 2. Experimental Data and Preprocessing

We investigate metabolite profiles (GC-MS data) of early development of *Arabidopsis thaliana*. More precisely, metabolite profiles of plants of the two homozygous lines C24 and Columbia (Col-0: depicted as Col in what follows) and the reciprocal crosses ColxC24 and C24xCol are studied. Metabolite profiles of the two homozygous genotypes C24xC24 and ColxCol and the two heterozygous genotypes C24xCol and ColxC24 were measured at 7 time points (0, 12, 24, 36, 48, 72, and 96 hours after sowing (HAS)). For each measurement, a Petri dish of seedlings was grown and fully harvested after the specific time of growing. In our balanced cross-factorial design, four replicates were assessed per genotype and time point, measured at three different measuring days, such that each genotype time point combination was measured at least once per measuring day. The raw data preparation was performed as in [25]; afterwards, the data were log-transformed. Overall, 210 metabolites have been measured. Eight of them contained more than 20% missing values, and were therefore excluded from further analysis.

For normalization, we chose a linear modeling approach, involving the factors "C24xC24," "ColxCol," "C24xCol," "ColxC24" denoting the four genotypes, the factor  denoting the 7 time points of the developmental time series, their interaction , as well as factor  denoting the measuring day. The linear regression was fit on a per-metabolite basis for the following model, for which , the logarithm of the raw metabolite signal, is modeled as being dependent on the factors described above: (1)

Here,  gives the overall mean, and the four genotypes are denoted with index , the seven time points with index , the measuring days with index , and the replicates with index . Normalized metabolite profiles were obtained using the effect estimates from the fit of model (1) as in (2). This way, data were corrected for measuring day effects and correct mean values were calculated, even for combinations with single missing values: (2)

**Figure 2:**
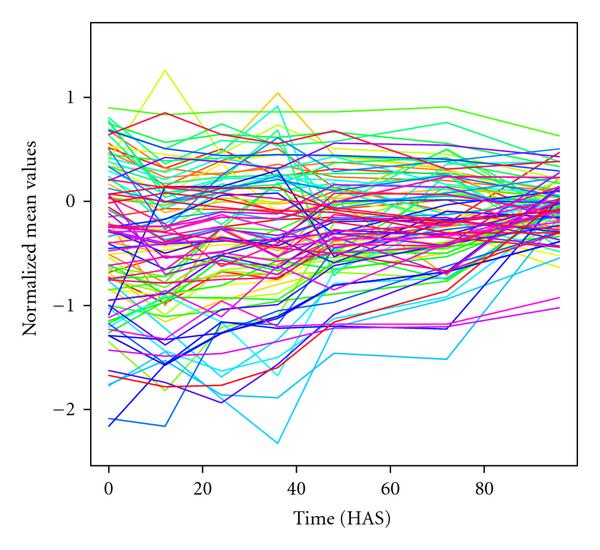
**Profiles of normalized values for each metabolite (202 different colors) over seven points for the genotype C24xC24 as obtained from (2)**.

The resulting time series of normalized metabolite profiles is plotted in Figure 2 for genotype C24xC24.

## 3. Methods

### 3.1. Modeling and Simulation

Our conceptual modeling approach employs a model of association to simulate adaptability in regulatory networks. Adaptedness can be described as the ability to give a correct response (output) to an environmental or developmental challenge (input). Hence, an adaptation can be viewed as the correct *association* of a response to the input in question. Correspondingly, adaptability is the number of differentiated correct adaptations that a regulatory system is able to realize.

**Figure 3:**
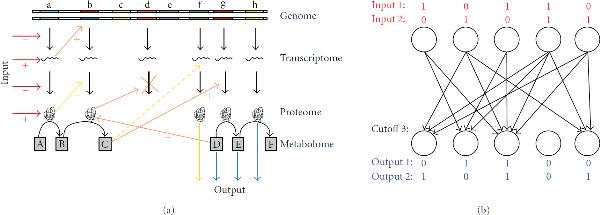
**Schematic representation of molecular networks (a) with *synthesis* (black arrows) and *regulatory* functions (colored arrows), as homomorphic to the association network model (b), representing a two-layer feed-forward Steinbuch matrix**. Associated input-output pairs are depicted in corresponding colors (blue and red). Black arrows depict regulatory interactions between specific input and output nodes.

Figure 3(a) shows a scheme representing a diploid genome and various levels of gene expression (transcriptome, proteome, and metabolome). Black arrows represent *synthesis*, and colored arrows symbolize *regulatory functions*. Simplifying this scheme leads to the simplest possible homomorphic model—an association matrix as in Figure 3(b). Here, input and output are associated via the interactions between input layer and output layer. In the output layer, signals from the input layer are summed up and compared to a threshold cutoff as to yield an output of "1" if larger or equal, or of "0" if smaller. The association network can be modeled mathematically as an  matrix , where  denotes the size of the network which is given by the number of nodes in the input and output layers, respectively (e.g.,  for the network in Figure 3(b)). In this model, each molecular entity (metabolite, protein, or transcript) has two possible states: "0" or "1." The input signal  is converted into the output  through (3)

where  is a threshold function that is applied componentwise: (4)

where, for example, .

For the case given in Figure 3(b), the matrix for the association network is given by (5)

We conducted a small simulation study, employing an association matrix of size  which is capable of correctly associating 4 pairs of input-output vectors. The model was trained to reproduce these predefined input-output pairs, which can be interpreted as some kind of crucial regulatory reply (regulatory step) to cope with a special environmental challenge. The study should reveal whether a partial correlation analysis of state profiles for the nodes of the network is a valid possibility to study the causal regulatory interactions in this network. 100 randomly generated input vectors  and their corresponding outputs  were stored as profile data and partial correlations calculated as detailed in what follows.

### 3.2. Network Statistics

Different types of networks can be used to assess the underlying biochemical interaction network from high-throughput metabolomic data. For our analysis, we have used partial correlations. This belonging network is known as graphical Gaussian model (GGM), concentration graph, covariance selection graph, conditional independent graph (CIG), or Markov random field [26]. Partial correlations have been shown to be a suitable method for deducing regulatory interactions from observational (noninterventional) data [27]. They are calculated by Opgen-Rhein and Strimmer [26] from metabolite levels as in (6)

The bases for these values are the normalized metabolite values for the seven time points from (2) for each genotype and each of the analyzed 202 metabolites. Thus, for any two metabolites of one of the four genotypes, partial correlations can be calculated based on the seven pairs of metabolite values corresponding to the seven time points.  is the estimate of the partial correlation between the metabolites  and .  are the elements of the inverse covariance matrix which is estimated using a shrinkage estimator [28]. The algorithm is implemented in the  package *GeneNet* [29].

We investigate changes for the partial correlation structure between heterozygous and homozygous genotypes by first calculating a "midparent" value as mean value for each metabolite and both homozygous genotypes: (7)

for all metabolites .

Second, the heterosis effects were calculated for both heterozygotes as increase of absolute partial correlation in the heterozygote compared to the midparent value. These values were calculated for all pairwise combinations of metabolites (see (8) and compare to Figure 1(a)). We considered absolute correlations because an increase of positive correlations should be equally weighted as a decrease of a negative correlation: (8)

Here,  denotes the respective heterozygous line ().

Third, to characterize changes in partial correlation with respect to the midparent value on a per-metabolite basis, for each metabolite  we calculated the mean values across all pairs involving this metabolite: (9)

Distributions of  were displayed and compared.

To investigate if the metabolites showing the largest values for  had a specific distribution over metabolite pathways, we visualized the first thirty metabolites in a ranking of  for each heterozygous line using MAPMAN [30]. MAPMAN is a tool to display large datasets onto diagrams of metabolic pathways.

Not only global distributions of changes in partial correlations but also structural properties of partial correlation networks could be different between homozygous and heterozygous lines. In such networks, *edges* are significant partial correlations, computed according to Opgen-Rhein and Strimmer [26]. *P*-values were corrected using the FDR correction described by Benjamini and Hochberg [31]. Accordingly, *nodes* in partial correlation networks are the metabolites contributing to significant partial correlations.

The *degree* of such a node is defined as the number of edges it is part of. We characterized the partial correlation networks of the two homozygous and the two heterozygous lines by counting significant edges and the participating nodes, as well as calculating the mean degree values over all nodes of a network.

## 4. Results

### 4.1. Simulation Results

**Figure 4:**
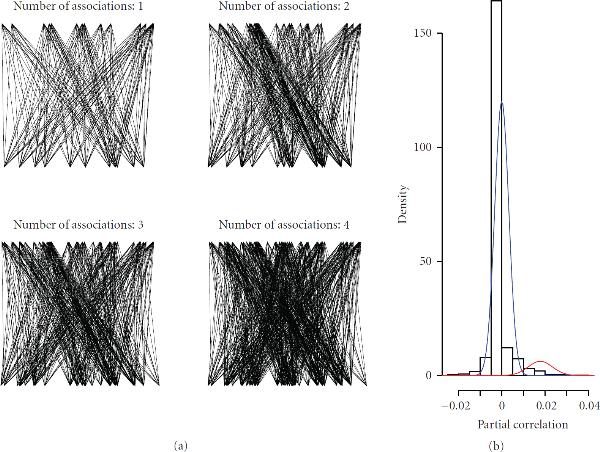
**Example for a  Steinbuch matrix**. (a) Increase in number of regulatory interactions between input and output layers, representing an increasing number of association pairs. (b) Analysis of the matrix of A with the ability to reproduce 4 predefined association pairs. Distribution of partial correlations for noninteracting input-output nodes (blue: entry "0" in ) and for interacting input-output nodes (red: entry "1" in ).

When comparing association matrices capable of reproducing an increasing number of associations (), the belonging networks show an increasing number of causal interactions between input and output layers (see Figure 4(a)).

Our small simulation study, where we recorded outputs for 100 random inputs to a  association matrix reproducing 4 input-output associations, revealed that causal interactions between input and output layers lead to increased partial correlations of the respective nodes. As demonstrated in Figure 4(b), for our model, causal interactions can be deduced from observational profile data by calculating partial correlations. These properties of our conceptual model led to the development of a network structure-based model of heterosis as outlined in what follows.

### 4.2. Network Hypothesis of Heterosis

As suggested by Robertson and Reeve [15], heterozygotes are likely to possess a greater biochemical versatility by carrying a greater diversity of alleles. Heterosis would then result from a reduced sensitivity to environmental variations since there will be ways of overcoming such challenges. In other words, the heterosis phenomenon may be due to higher adaptability in heterozygotes.

Correspondingly, as illustrated in Figure 3(a), the molecular network of a heterozygous cross may contain a proportion of heterozygous loci, as for gene "b," for example. The additional alleles at this locus may lead to *additional* regulatory interactions in the molecular network (yellow arrows in Figure 3(a)). In our model, as shown in the simulation (see Figure 4(b)), additional causal interactions are the basis of an increasing number of associations in the repertoire of the Steinbuch network. It is known from earlier studies of system properties of the Steinbuch network that there exists a *limit of associated pairs* for a network of a given size [32]. A Steinbuch network of a given size can be built to be able to differentiate between a certain number of inputs by "responding" with the (associated) belonging outputs, and not more. This is a known system property of this type of regulatory networks—but also for other types of neural networks. Moreover, if we measure an increasing amount of partial correlations within a molecular network, this might correlate with an increased amount of regulatory "challenge-response" pairs managed by this network, and hence with increased adaptability. Interpreting these properties as conceptual model for adaptation and adaptability in molecular regulatory networks leads to two predictions for the case of heterosis.

(1) There should exist a limit for increasing hybrid vigor with increasing level of heterozygosity. Increasing the genetic distance of homozygous parental lines beyond a certain threshold should result in less hybrid vigor if these parental lines are genetically too different. When mating two similar homozygous genotypes, only few additional regulatory connections within the molecular networks can be expected. However, when mating homozygous genotypes which are genetically very different (with large genetic distance), the limit of the resulting merged molecular network structures may be exceeded—in the sense that regulatory interactions in the network of the resulting heterozygotes do not match and therefore do not lead to additional possibilities of valid regulatory answers.

(2) Molecular interactions in regulatory networks of heterozygotes should be slightly enriched. This increased number of "challenge-response" pairs is modeled as a higher number of association pairs in our conceptual model, interpretable as increased adaptability leading to heterosis. As for the model, where we were able to measure interactions as increased partial correlations, we also expect an increase in partial correlations from homozygotes to heterozygotes for the experimentally observed dynamics of biological regulatory networks.

**Figure 5:**
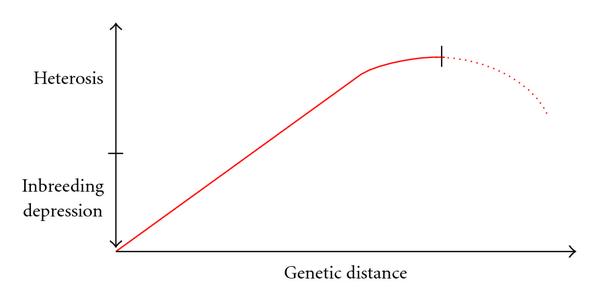
**Possible relationship between genetic distance of the parental lines and hybrid vigor in the offspring**. There is evidence for the existence of a limit of increase in hybrid vigor, as indicated in [33–36].

For evaluating prediction 1, we had no own experimental data, as these were only based on crosses of two homozygous lines. Instead, we analyzed the literature basis of a possible relationship between heterosis and genetic diversity. Figure 5 summarizes this literature view regarding a possible limit of gain in hybrid vigor in offspring for increasing genetic diversity between the parental lines. From studies in maize as well as beans, it likely seems that, with increasing genetic diversity between the parental lines, the resulting hybrid vigor for the offspring at first increases. However, for parental lines which are genetically too different, it is expected to decrease again [33–36]. We want to emphasize that, given the literature basis as investigated, further research on the first part of our network hypothesis of heterosis seems promising and necessary as at the moment we cannot draw stronger conclusions.

Regarding prediction 2, we studied our experimental dataset, the *Arabidopsis* metabolome of a developmental time series (see Section 4.3). From the perspective of our model, Figure 3(a) illustrates how the molecular network of heterozygotes contains additional regulatory possibilities. In the association network model, these correspond to additional connections (interactions) between input and output layers, enabling the network to add additional associations to its repertoire. These additional associations (input-output pairs) represent a grown repertoire of adaptations, or increased adaptability, enabling increased hybrid vigor. The objective of our experimental data analyses was to investigate if such increase in molecular interactions would be measurable as increase in partial correlations as a global network property for the metabolite profiles recorded during *Arabidopsis* development.

### 4.3. Analysis of Experimental Data

Our experimental data were metabolite profiles from development of *Arabidopsis thaliana* (see Figure 2). To test our hypothesis that heterosis comes as increasing adaptability and should result in increasing connectivity of molecular networks, we had first conducted a small simulation study (see Section 4.1). Its findings provide the basis for our investigation of partial correlation structures of the metabolomes of heterozygous and homozygous genotypes for the experimental data, as we want to test a hypothesis about increased regulatory possibilities in heterozygotes and the belonging structures of molecular profiles. Hence, we analyzed partial correlations according to Opgen-Rhein and Strimmer [26] for our experimental dataset.

**Figure 6:**
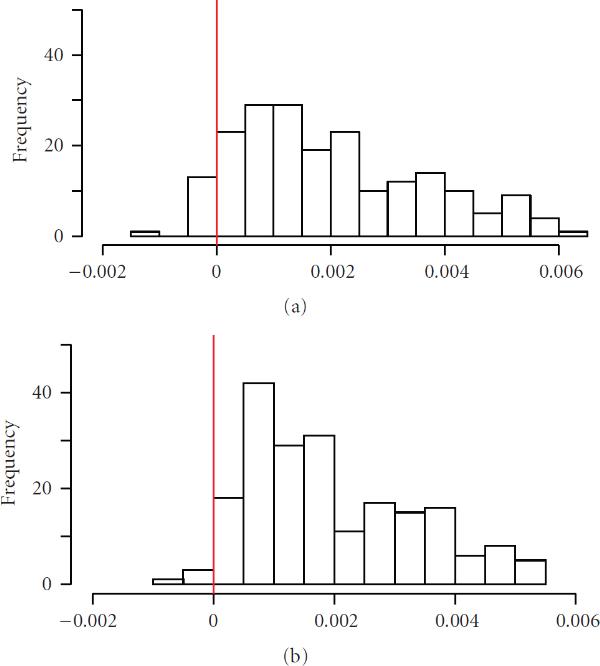
Display of  for  (see (9)). The mean differences for most metabolites between the partial correlations of genotype C24xCol (a) as well as genotype ColxC24 (b) to the average of the homozygotes (midparent) are positive values.

**Figure 7:**
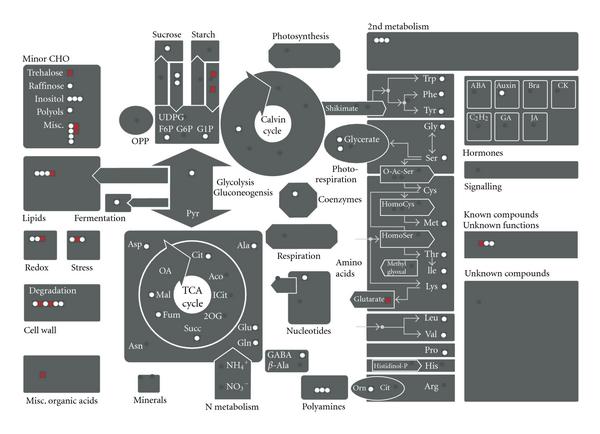
**Metabolites with highest mean differences between absolute partial correlation values of genotype C24xCol and the mean of the homozygous lines are displayed on plant biochemical pathways (red)**. White: metabolites that are present in the MAPMAN [30] annotation list as well as in our metabolite list but not within the top 30 list. Dark gray: not measured.

The average heterosis increase of the partial correlations in the heterozygous lines as compared to the midparent value (mean of the homozygous lines) was calculated (; see (9)). Results are displayed in Figure 6. The histograms for  for the genotype C24xCol (Figure 6(a)) as well as  for the genotype ColxC24 (Figure 6(b)) show that for a majority of the metabolites the calculated difference is positive. This means that the mean partial correlation values of either heterozygous genotype are larger than the average of the homozygotes (midparent). For each heterozygous genotype, the 30 metabolites that show the largest difference were determined. For the genotype C24xCol, these selected metabolites are displayed onto a diagram of biochemical pathways in Figure 7 using MAPMAN [30] to study possible pathway-related differences in the partial correlation values between homozygous and heterozygous genotypes. Metabolites of the top 30 are marked as red points. The picture does not contain 30 red points because the top 30 list contains several unknown metabolites. Furthermore, not all metabolites are available in the MAPMAN annotation. The displayed metabolites are relatively evenly distributed over all illustrated pathways. For the genotype ColxC24, this distribution looks similar (data not shown). Twelve metabolites were in common for the top 30 lists of both heterozygous genotypes.

In Table 1, the detailed results of the connectivity analysis are listed. For all metabolites, the partial correlations are based on the time series of the 7 time points from 0 HAS to 96 HAS. In Table 1, the number of significant edges and the number of nodes (metabolites) that belong to these edges are shown. Our main focus in this analysis was on mean degree. These mean degree values were calculated on the basis of the number of nodes with significant edges (see definition at the end of Section 3.2).

**Table 1:** Significant partial correlations (significance level: ).

Genotype	No. of significant edges	Corresponding nodes	Mean degree
C24xC24	10	13	1.54
ColxCol	23	23	2
C24xCol	81	45	3.60
ColxC24	64	40	3.20

**Figure 8:**
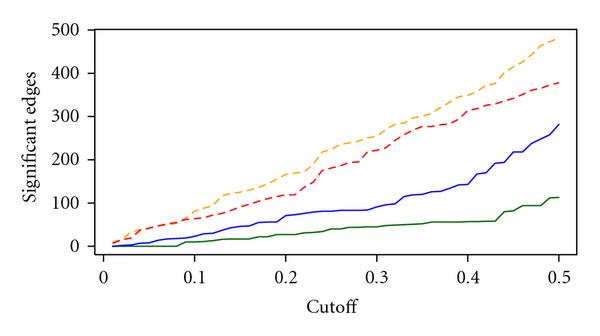
**Display of numbers of detected significant partial correlations as being dependent on corrected -value cutoff (significant partial correlations) for the 4 genotypes**. Heterozygotes (dashed lines) show a higher number of significant edges throughout. (C24xC24: green; ColxCol: blue; C24xCol: orange; ColxC24: red).

Both homozygous genotypes show lower mean degrees than either heterozygote. As shown in Figure 8, the relation between the numbers of significant edges of the heterozygotes and those of the homozygotes is nearly independent of the cutoff used.

**Figure 9:**
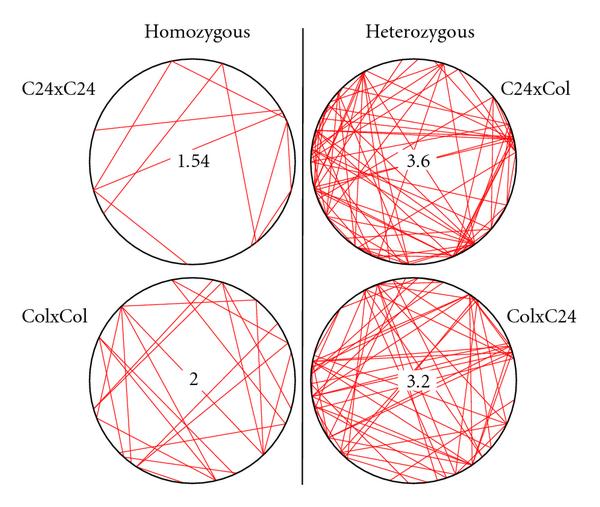
**Connection plots based on partial correlations, using a cutoff  for the belonging FDR-corrected *P*-values**. The heterozygous genotypes show more significant edges and a higher connectivity than the homozygous genotypes. Mean degrees are given for each genotype.

We choose a cutoff  for the FDR-corrected *P*-value to determine the significant edges in each analysis. This outcome is illustrated in Figure 9. The partial correlation networks of the two heterozygous genotypes show more connections than the networks of the homozygous genotypes.

Hence, results of Figures 6 and 9 point towards the same tendency, supporting the "increasing-connectivity" prediction of our network hypothesis of heterosis. This tendency is strengthened as most of the 30 metabolites that show the largest differences between the heterozygotes and the midparent value also have significant edges. In more detail, for genotype C24xCol, 25 of the top 30 metabolites and, for genotype ColxC24, 27 of the top 30 metabolites have significant edges. Total numbers of nodes with significant edges are 45 and 40, respectively (see Table 1). On average, for either heterozygous genotype, 86.7% of the top 30 metabolites show significant edges.

## 5. Discussion

We have developed a network structure-based hypothesis of heterosis. It is a systems biological approach to relate biological function to molecular network structure. Our hypothesis results in the following predictions. First, system properties of our network modeling approach suggest the existence of an upper limit for the heterosis effect when genetic distance of crossed homozygous parental lines becomes too large. Second, molecular networks of heterozygotes should contain additional interactions compared to those of their homozygous parents. These additional interactions should lead to increased partial correlations in molecular networks of heterozygotes. For the first prediction, we found support in the literature suggesting an upper limit for the heterosis effect. However, as we do not have sufficient additional own experimental evidence, no final conclusion can be drawn for this case. Further investigations seem promising and necessary. Regarding the prediction of increased connectivity of molecular networks in heterozygotes, for our own experimental metabolome dataset of *Arabidopsis,* such increased connectivity was observable for both heterozygous crosses. It was this phase of early *Arabidopsis* development in which the heterosis effect is established. The predicted pattern is visible for the majority of metabolites. However, also for the second part of our network hypothesis of heterosis, we call for additional experimental evidence, preferably on additional levels of molecular regulatory networks, such as proteomics or transcriptome data. To summarize, we present a conceptual frame for explaining the heterosis phenomenon from a molecular network perspective together with two hypotheses and their predictions, for which we were able to find the first supporting evidence from the literature and own experimental data.

We are convinced that research towards understanding the biological phenomenon of heterosis can particularly gain from a systems biological approach focused on *interactions* of molecular building blocks and global structures of molecular biological networks. Towards elucidating the genetic basis of heterosis, Melchinger et al. [37] have already shown that, taking a statistical modeling approach, epistatic interactions of individual loci with the entire genetical background constitute a major component of genetic variation important to explain heterosis. However, the mapping of interaction terms in models of quantitative genetics to structures in molecular regulatory networks is nontrivial [19,38]. Our approach to investigate global network structures in molecular interaction networks for this reason is to be taken as *complementary* to the quantitative genetics view.

Meyer et al. [24] report for *Arabidopsis thaliana* development that it is the *early* phase of development (till one week of seedlings' growth) during which the heterosis phenotype for biomass is established. In later phases of the plant life, relative differences between heterozygotes and homozygotes are not further growing. The first observation coincides with our results. We observe increased connectivity in partial correlation networks during this period of development. It would be interesting to see—this is planned as future experimental study—if during the later phase, when according to [24] biomass heterosis is visible but no longer increasing, there is no indication of increased connectivity in the metabolome any longer. 

The majority of metabolites investigated showed an increase in interaction connectivities. We tried to find common functionalities for the top 30 metabolites with most obvious changes. However, we were not able to detect evidence towards an accumulation of such metabolites within certain pathways or modules (MAPMAN categories). We hypothesize that it may be these metabolites that during the early phase of *Arabidopsis* development are mainly involved in *regulatory interactions*—to enable adaptation to the climatic chamber during the first contact with this environment.

Only part of the observed changes in partial correlations between heterozygous lines and the midparent value of both homozygotes can be based upon *significant* partial correlations (compare Figures 6 and 9). However, the same tendency is apparent for the global view as well as for the restriction to significant correlations. It is the sparsely designed experimental data that does not allow for a more precise analysis. Seven time points are clearly the *lower limit* of correlation analyses involving around two hundred metabolite species. We look forward to more generously designed experiments for testing our network structure-based hypotheses for heterosis.

Our modeling approach is *conceptual* as advocated for by, for example, Wissel [20] and Shubik [21]. It builds upon the understanding of the heterosis phenomenon as increased adaptability. This understanding has its roots already at the beginning of the 20th century in maize genetics [14] and since then has been expressed also within the context of hybrid vigor observed for other plant species as well as model animals (see, e.g., [15,39,40]). We make use of a model for adaptability which was originally designed to model associative memory (the Steinbuch matrix) [23]. Within our model, being *adapted* means to respond in a correct way when confronted with a certain environmental or developmental stimulus, while *adaptability* means the potential to respond to a number of different stimuli with differentiated correct responses. The simplicity of this conceptual modeling implies rather *general* predictions. In our case, these are the limit-of-heterosis increase prediction and the increasing-connectivity prediction. These are predicted for a huge class of interaction networks, independent of molecular species. Motif analyses in different molecular interaction networks as well as within organisms of different kingdoms (prokaryotes and eukaryotes) have shown that certain motifs are always present. The "multi-input motif" is a prominent example. Here, we refer to the work by Milo et al. [10] and Lee et al. [12]. The *multi-input motif* has the same structure as our association network model, which was first proposed in 1961 by Steinbuch [23]. Furthermore, molecular interactions are often modeled based on a sigmoidal relationship as approximated by the boolean kind of interaction in the Steinbuch model (discussed in [41]).

A central assumption underlying motif analyses as well as our modeling approach for this work is that neglecting the diversity of different kinds of molecular species that interact within real molecular networks does not cause harm at the rather general level of conclusions of our conceptual investigations. It is clear that natural molecular networks cannot be reduced to a very simplistic model in *all* their structural and dynamical properties. However, we chose to follow Shubik's call for the most *parsimonious* modeling approach [21]. Also, heterosis is a very general biological phenomenon together with its counterpart inbreeding depression. Both phenomena are occurring over a broad variety of sexually reproducing organisms. For this reason, approaches towards understanding the systems biological foundations of these phenomena should be independent of all organism-specific parameters, in other words as simple as possible.

Choosing the metabolome level, as in our study, is just one possibility. With [5], we argue that the *extended regulatory network* of an organism can be mapped to any of its levels of gene expression ("omics" levels). However, the modeler has to be aware of all possible hidden variables constituting each of the investigated interactions. These hidden variables are representations of the molecules from the "omics" levels which were not modeled. In our case, for example, regulatory interactions between metabolites have no direct correspondence to metabolic pathways. Moreover, as is true for gene expression studies for the case of transcription factors, also in metabolomics it is not at all possible to assess *all* molecules, but only a small fraction. The measurable fraction may or may not be a biased sample from the entire metabolome, and for this reason inferring network structures from such a sample has always to be taken with care (for an example concerning network statistics in protein interaction networks, see [42]). Also, we are aware of the problem of cell-type heterogeneity in our samples which are basically whole embryo/plant homogenates. Measured profiles in our case represent metabolite levels of the major cell type. In addition, it is important to take into account the fact that those 202 metabolites in our investigation are just around 10% (possibly less) of the metabolites that are supposed to be present in *Arabidopsis thaliana* [43]. Thus, our network structure-based hypothesis of heterosis was validated only for the core carbon metabolism. These small molecules (e.g., sugars, amino acids, and carbon acids) act mostly within energy metabolism and as precursors for building the larger biomolecules, proteins, and nucleic and fatty acids. These metabolites represent what is currently measurable with the GC-MS metabolite profiling experiments.

For future investigations of molecular network structures with respect to the heterosis phenomenon, it will be an interesting challenge to extend the time series design of the current study in several aspects. To enable a more general conclusion regarding the two predictions from our network hypothesis of heterosis, it would be worth comparing several different homozygous lines and their reciprocal offspring. Also, genetically very different lines should be included to approach a direct test of the limit-of-heterosis increase prediction. Moreover, time points should be set more dense (e.g., as 10-hour intervals) and over a longer time scale (e.g., at least along the first four weeks of *Arabidopsis thaliana* development). Such a design would enable a higher precision for both estimating partial correlation structures as well as assessing a possible change of such structures during later phases of development, for which according to Meyer et al. [24] no additional heterosis effects are arising. Furthermore, studies are already planned to analyze *transcript data* measured under the same conditions as our metabolome dataset. This would enable us to show, first, how two levels of the extended regulatory network act together taking an integrative bioinformatics approach (see, e.g., [44]). Second, it would be possible to test the increasing-connectivity prediction of heterosis also for the level of the transcriptome.

Regarding alternative approaches to measure differential network structures in molecular networks of homozygotes and heterozygotes, there exist a number of possible choices. An alternative type of networks used for inference of biochemical interaction networks is, for example, the so-called relevance network. Butte et al. [45] base their method on a pairwise Pearson correlation of all features. A serious limitation of relevance networks is that they contain many indirect correlations because they cannot distinguish between direct and indirect interactions. For our kind of *observational* data, Werhli et al. have shown that it is preferable to use association networks to infer regulatory interactions [27]. For this reason, we decided to analyze partial correlations as proposed by Opgen-Rhein and Strimmer [26]. We also favored the regularized inference of the covariance matrix they proposed, which is applicable for data with a small sample size and a comparatively large number of variables, as in our metabolome dataset. Our simulation study was able to demonstrate that, when observing a number of partial correlations from the Steinbuch model, these could be used to identify the nodes of input and output layers which were connected in the regulatory architecture of the network model to reproduce four predefined input-output patterns. Hence, for our conceptual model, regulatory interactions could be deduced from partial correlations. A possibly promising way to extend our analyses could be oriented along the lines of the work by Saul and Filkov [11] who proposed to use the so-called exponential random graph models. They demonstrate their utility in modeling the architecture of biological networks as a function of a number of different measures of local network structure, not only a single measure as in our case. The flexibility, in terms of the number of available local feature choices, and scalability possibly make this approach a suitable alternative for statistical modeling of biological networks.

To summarize, in our work we followed the call of Barabási and Oltvai [46] who conclude their review on *network biology* by stating that structure, topology, network usage, robustness, and function are deeply interlinked, forcing us to complement the "local" molecule-based research with integrated approaches that address the properties of regulatory networks at a systems biological level. In our study, we have done so by proposing a network structure-based model of heterosis and investigating its predictions for an experimental omics dataset. Heterotic phenotypes of *Arabidopsis* are mirrored as increased connectivity in metabolome partial correlation networks. A limit of hybrid vigor increase for increasing genetic distance of crossed parents is also correctly predicted. These results hold for the measured part of the metabolome, mostly central carbon metabolism.

Our conclusions cannot be more than an illustrative example of how a hypothesis can be built about a possible relation of biological network structure to biological function (in our case, the heterosis phenomenon). We advertise our approach as a way of investigating heterosis complementary to the quantitative genetics approach, and look forward to future unifying approaches to these two fields.
